# Atopic dermatitis: Is innate or adaptive immunity in control? A clinical perspective

**DOI:** 10.3389/fimmu.2022.943640

**Published:** 2022-07-27

**Authors:** Peck Y. Ong

**Affiliations:** Division of Clinical Immunology and Allergy, Children’s Hospital Los Angeles, Department of Pediatrics, Keck School of Medicine, University of Southern California, Los Angeles, CA, United States

**Keywords:** atopic dermatitis, *S. aureus*, *S. epiderimidis*, coagulase - negative *Staphylococcus*, IL-17A

## Abstract

Atopic dermatitis (AD) is a chronic inflammatory skin disease with barrier defects and immune dysregulations. The pathogenesis of AD involves the physical barrier as well as epithelial cells, which are considered a vital part of the innate immunity of the skin. The importance of filaggrin mutations in the pathogenesis of AD has also been well-established with reproducible results around the world in multiple studies and ethnic groups. This protein plays an important role in skin barrier functions and further reaffirms barrier defects as one of the primary causes of AD. The main epithelial cells, keratinocytes, function as a major sentinel for the skin in detecting danger signals or microbial pathogens, and trigger downstream immune responses. In AD, these cells express TSLP, IL-33 and IL-25, which lead to downstream systemic production of type 2 cytokines. In spite of major advances in our understanding of the innate immunity of AD, recent success in the systemic therapeutics of AD have focused on targeting the products of the adaptive immunity, particularly cytokines produced by T cells. In addition to type 2 cytokines, type 17 cytokines have also been implicated in the pathogenesis of AD. The current review examines the implications of these cytokines in AD from clinical perspectives.

## Introduction

Atopic dermatitis (AD) is a chronic inflammatory skin disease that typically presents in early childhood. It is characterized by dry, erythematous and pruritic skin rashes. These rashes lead to a vicious cycle of itching and scratching that eventually disrupts sleep and daily activities. The poor quality of life in AD patients, as compared to other chronic conditions, has been well-documented (1). Patients with AD are conscious about their rashes and may be bullied or discriminated by peers due to their appearance (2). Adult patients may make life and career decisions based on their AD (3). Family members of AD patients are also affected due to the care and attention that are needed in these patients (4). Other co-morbidities of AD include depression, anxiety disorder, infections and other atopic diseases including food allergy, asthma and allergic rhinitis.

## Cytokine-targeted therapy and mechanisms of atopic dermatitis

Looking back 20 years in our understanding of AD pathogenesis (5), which highlighted the importance of cell-mediated immunity, the current review aims to re-examine what we have learnt from the recent development in the treatments of AD. Products of cell-mediated immunity including type 2 cytokines, IL-4, IL-5 and IL-13, and type 1 cytokine, IFN-γ, were thought to be important in the acute and chronic lesions of AD, respectively. Clinical studies with anti-IL-5 monoclonal antibody (mAb) and IFN-γ in AD patients have either been ineffective or inconclusive (6). On the other hand, targeting both IL-4 and IL-13 has revolutionized the treatment of moderate to severe AD. The efficacy of blocking both IL-4 and IL-13 by dupilumab, which targets the IL-4α receptor, has been shown in both phase 2 and 3 trials (7, 8). The observation in real-world setting has further confirmed the efficacy of dupilumab (9), and established the role of IL-4 and IL-13 in the pathogenesis of AD. The approval of dupilumab led the way to more systemic treatments for AD that aim to target type 2 cytokines or type 2-associated cytokines such as IL-31. These recently-approved treatments include anti-IL-13 mAb and oral Janus kinase (JAK) inhibitors (10).

Th2 cells were thought to be the main source of IL-4 and IL-13 (5). More recent studies have uncovered other cells that are capable of producing these two cytokines and other cytokines that enhance the production of IL-4 and IL-13 (11). In addition to IL-4 and IL-13, an increased expression of IL-17A and its potential pathogenic role has also become an emerging concept in AD (12, 13). To further understand the origin of these cytokines in AD, a basic understanding of the immune system in relation to AD is needed.

## An overview of skin immunity

“The immune system clearly exists to protect the host from infectious agents” (14). Janeway’s deduction of the innate immune response being specific for infectious non-self led to his postulation of pattern-recognition receptors and eventual discovery of toll-like receptors (TLRs) (15). These receptors act as the immune system’s sentinel for invading microbial pathogens and initiate immune response against these organisms. A classic pathway of immune response at the mucosal surface is the activation of epithelial cells by TLR, leading to the production of chemokines, which summon antigen-presenting cells and effector T cells to the site of infection (16). IFN-γ production by Th1 cells has long known to be an important mediator of cellular immunity. The importance of type 17 immunity is illustrated by STAT3 mutation in autosomal dominant hyper-IgE syndrome, which results in a lack of Th17 cell development and increased susceptibility to skin bacterial infections and other mucosal fungal opportunistic infections such as oral candidiasis and pneumocystis lung infections (17, 18). Other contributors of type 17 immunity in the skin include innate γδ T cells and innate lymphoid cell group 3 (ILC3) (19, 20). Much of Janeway’s concept has been applied to study immune responses during an actual or impending infection. The mucosal barrier was thought to be simply a physical barrier and sensor of infectious threats. More recent understanding of barrier defense presents a more complex mechanism that involves more than just the epithelial cells, but also commensal microbes and adaptive immunity. The need for an efficient barrier is not only for prevention of infections, but also to prevent collateral damage from an effector response against an invading pathogen. Excessive type 1 and type 17 responses present as inflammation that can be devastating to the host, as seen in conditions such as psoriasis and other autoimmune conditions. While stratum corneum, corneocytes, keratinocytes and epidermal molecules including filaggrin, lipids and tight junctions serve as the physical barrier against the invasion of pathogens, any compromise to this physical barrier (eg. an inherent barrier defect such as filaggrin deficiency or an immature newborn skin) would put the skin at risk for infection and inflammation. The so-called homeostatic immunity involves the interaction between adaptive immunity and skin microbiota (21). It has emerged as an important concept in maintaining and repairing skin barrier. Beneath the skin barrier is the home for many memory T cells. Indeed, adult skin contains four times as many memory T cells as there are in peripheral blood (22). Among these memory T cells are a subset known as tissue-resident memory T cells (T_RM_), which are non-circulating T cells that are tasked to respond rapidly to invading pathogens (23). These pathogens are captured and processed by antigen-presenting cells, and presented to T cells bearing T cell receptors (TCRs) that are specific for the pathogens in the context of major histocompatibility complex (MHC) restriction. However, it is now known that not all TCRs among T_RM_ are specific for pathogens. Some of the TCRs are directed towards normal skin microbiota such as coagulase-negative staphylococci (CoNS). A subsets of T_RM_ that has emerged as the main commensal-specific T cells is the CD8+ T cells that express IL-17A (Tc17) (24). TCRs on *S. epidermidis*-specific Tc17 are restricted to unconventional MHC class Ib molecule, rather than the classic MHC Ia molecule for CD8+ T cells. Of interest, the peptides that are recognized by these Tc17 cells come from secreted proteins of the commensal bacteria. One such peptide is N-formyl methionine from *S. epidermidis*. These *S. epidermidis*-specific Tc17 were found to express a panel of genes that are involved in tissue repair. They were also shown to promote and accelerate wound healing. Harrison et al. (25) subsequently found that *S. epidermidis*-specific Tc17 are capable of co-expressing the transcription factors retinoic acid-related orphan receptor-γt (RORγt) for type 17 immunity as well as GATA-binding protein 3 (GATA-3) for type 2 immunity (25). They found that these Tc17 cells express IL-5 and IL-13 mRNA without producing IL-5 and IL-13 proteins. On skin barrier breach and an encounter between these specific Tc17 and *S. epidermidis* leads to the production of IL-5 and IL-13 in the presence of IL-18. As type 2 immunity has been implicated to play a role in barrier repair (26), the study by Harrison et al. demonstrates the plasticity of Tc17 to switch between type 17 immunity against invading pathogens and type 2 immunity for barrier repair. Mucosal-associated invariant T (MAIT) cells are T cells expressing invariant αβ TCR that also recognizes processed peptides in the context of MHC class Ib (27). A classic example of these peptides are microbial-derived peptides from riboflavin (vitamin B12) (28). MAIT cells develop in a specific time window during the neonatal period when exposed to riboflavin-synthesizing bacteria (27). Cutaneous MAIT cells express IL-17A but not interferon-γ. These cells display IL-23R and their development can be promoted by IL-23 in the presence of riboflavin-producing bacteria. However, riboflavin-synthesizing *S. epidermidis*-specific MAIT cells expand in the presence of IL-18, rather than IL-23 (27). MAIT cells regulate response to tissue injury and promote tissue repair.

## Pathogenesis of atopic dermatitis

**Figure 1:**
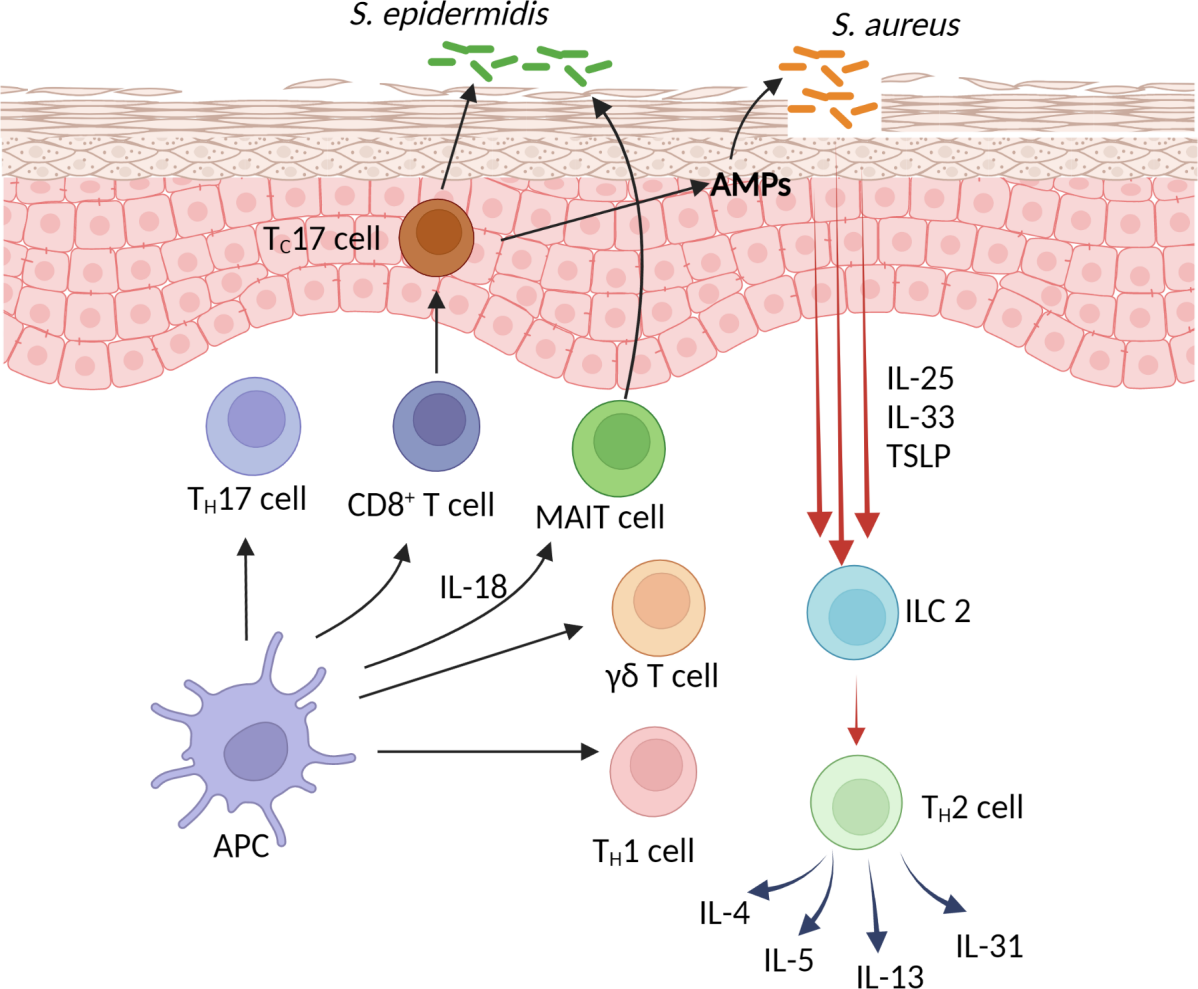
Skin barrier defense and the pathogenesis of atopic dermatitis.

One of the primary defects of AD is skin barrier defects. Normal-looking AD skin has been shown to be deficient in cholesterol, fatty acid, ceramides, filaggrin and tight junctions (29, 30). The observation that AD skin is deficient in filaggrin led to the eventual confirmation of filaggrin loss-of-function mutations in AD patients (31, 32). This establishes genetic barrier defects as one of the primary causes of AD. Another well-established genetic cause of AD is atopy. Parental history of atopic diseases including allergic rhinitis or asthma are known risk factors for AD (33). AD lesions are characterized by an increased expression of type 2 cytokines including IL-4, IL-5 and IL-13 (34, 35). Normal-looking AD skin also contains subclinical inflammation with increased expression of type 2 cytokines, as compared to healthy skin (34). AD keratinocytes express an increased level TSLP, IL-33 and IL-25 (36–38). These cytokines act as alarmins that induce the production of IL-4, IL-5 and IL-13 from ILC2 and Th2 cells (**Figure 1**). The role of Th22 cells in AD remains to be defined. A subset of CD1a-restricted Th22 cells reside primarily in the skin (39). These cells recognize self-lipid antigen presented on CD1a or CD1a itself independent of lipid antigen (39, 40). Their expression of IL-22 likely plays a role in epithelial repair mechanisms (40). TSLP also induces Th2-expression of IL-31, which has been shown to play a major role in the itch of AD (41). The pathogenic role of IL-4 and IL-13 in AD has now been well-supported by the resolution of clinical inflammation with the use of dupilumab in moderate to severe AD patients. In addition to causing inflammation, the chronic expression of these cytokines in AD lesions is a predisposing factor for increased *Staphylococcus aureus* (*S. aureus*) colonization and infections in these patients. This has also been supported by the use of dupilumab in AD patients in whom *S. aureus* colonization and clinical infections were shown to decrease significantly (42–44). Plausible mechanisms by which IL-4 and IL-13 contribute to *S. aureus* colonization and skin infections is through their suppressive effects on skin barrier functions, expression of antimicrobial peptides (AMPs) and type 17 immunity (45–47). Although the expression of IL-17A has consistently been found to be increased in AD lesions (48, 49), the role of this cytokine in AD pathogenesis is controversial. The use of monoclonal antibodies against IL-17A or IL-12/23, which inhibits the Th1 and Th17 pathways, have failed to improve AD disease severity (50–52). These observations argue against the role of IL-17A as part of AD inflammation, rather, its presence is part of an effector response against *S. aureus* (47, 53). Of interest, AD lesional IL-17A expression decreases after dupilumab treatment (54), further supporting its presence is in response to an increase in type 2 inflammation and *S. aureus*. While recurrent skin infections are common in AD patients, invasive staphylococcal infections are relatively rare and opportunistic fungal infections are absent in AD patients (55). The increased, yet attenuated, type 17 immunity in AD likely contributes to these protections. The role of CD8+ T cells in AD has been shown in mouse models (56, 57). CD4+ T cell-depleted AD lesions were predominated by IL-17-producing CD8+ T cells (57). In human AD lesions, an increased number of CD8+ T cells has also been described (58). Interestingly, most of these CD8+ T cells were found in the epidermis (58). There is a high percentage of IL-13-producing CD8+ T cells with a smaller number of IL-17A-producing CD8+ T cells in AD lesions (58). Whether these cells co-express type 2 and type 17 transcription factors, as described by Harrison et al. (25), will need further investigations. Further studies are also needed to analyze the specificity of their TCRs for CoNS or other cutaneous commensal bacteria. CD8+ MAIT cells have been found to be a source of IL-17A in human psoriatic lesions (59), whether these cells play a role in the barrier defense of AD will be also require further studies. The interaction between commensal-specific T cells and CoNS in barrier defense is dependent on the strains of CoNS as well as their mode of growth (biofilm *vs* planktonic) (60). Biofilm formation by *S. epidermidis* potentially inhibits the interaction between these bacteria and commensal-specific T cells, leading to a decreased production of IL-17A, therefore favoring *S. aureus* colonization (60). *S. aureus* virulence is also a major factor in AD inflammation. These bacteria are capable of inducing TSLP and IL-33, rather than AMPs, from keratinocytes of AD patients (61, 62). Superantigens produced by *S. aureus* also induce the expression IL-31 in AD patients (63). The underlying AD inflammation and clinical severity has been shown to be driven by differences in strain-specific *S. aureus* and *S. epidermidis* colonization (64).

## Conclusion

The pathogenesis of AD remains an intriguing topic over many decades. With the recent use of mAbs in the therapy of AD has broadened our understanding of the pathogenesis of this chronic skin condition. Type 2 cytokines, IL-4 and IL-13, not only contribute to AD inflammation, these 2 cytokines also predispose to *S. aureus* colonization and infections in AD patients. On the other hand, AD patients appear to have an intact type 17 immunity without contributing to AD inflammation. This is supported by an absence of rare opportunistic infections and a lack of efficacy by anti-IL-17A and anti-IL-12/23 therapies in the treatment of AD. While defects in skin barrier functions and innate immunity play an important role in the pathogenesis of AD, type 2 and type 17 cytokines have traditionally been thought to be contributed by Th2 and Th17 cells, respectively. However, recent concepts in mucosal immunity show that innate cells such as ILC2 and ILC3, and innate-like lymphocytes such as Tc17, MAIT cells and γδ T cells, which have both adaptive and innate properties that recognize nonclassical MHC Ib molecule, are also important contributors of type 2 and type 17 cytokines. Therefore, both innate and adaptive immunity likely contributes equally to the pathogenesis of AD.

## Author contributions

PO conceived the ideas, concepts, and wrote the paper.

## Acknowledgments

I thank Dr. Juli Wu for her assistance with **Figure 1**. PO is supported in part by The Albert and Bettie Sacchi Foundation.

## Conflict of interest

PO has been on advisory board for Incyte, Abbvie, Janssen, and has obtained research funding from Regeneron, Sanofi Genzyme, Leo, Incyte.

## Publisher’s note

All claims expressed in this article are solely those of the authors and do not necessarily represent those of their affiliated organizations, or those of the publisher, the editors and the reviewers. Any product that may be evaluated in this article, or claim that may be made by its manufacturer, is not guaranteed or endorsed by the publisher.
